# Fig seed oil improves intestinal damage caused by 5‐FU‐induced mucositis in rats

**DOI:** 10.1002/fsn3.4283

**Published:** 2024-06-18

**Authors:** Nurten Alan, Nazan Tuna Oran, Pınar Akokay Yılmaz, Aslı Çelik, Osman Yılmaz

**Affiliations:** ^1^ Department of Fundementals of Nursing, Faculty of Nursing Dokuz Eylül University Izmir Turkey; ^2^ Department of Midwifery, Faculty of Health Sciences Ege University Izmir Turkey; ^3^ Department of Medical Laboratory Kavram Vocational School Izmir Turkey; ^4^ Department of Dentistry Vocational School of Health Services, Dokuz Eylul University Izmir Turkey; ^5^ Department of Laboratory Animal Science, Health Sciences Institute Dokuz Eylül University Izmir Turkey

**Keywords:** chemotherapy, fig seed oil, fluorouracil, mucositis

## Abstract

Intestinal mucositis poses a significant concern associated with cancer therapy. This study aims to investigate the protective and/or healing effect of fig seed oil (FSO) on 5‐fluorouracil (5‐FU)‐induced intestinal mucositis by targeting inflammatory markers and histologic changes in rats. Albino Wistar adult rats were randomly divided into four groups, including three male and three female animals. All the animals in the four groups had a normal standard diet and water throughout the experimental period, which lasted up to 11 days. Rats were administered FSO 0.6 mL (mucositis FSO group) and FSO 0.2 mL (mucositis FSO‐R group) daily throughout the experiment. These two groups and one additional group (mucositis group) were given an intraperitoneal injection of 5‐FU (300 mg/kg) on Day 5 of the experiment. In contrast, the fourth group (Control group) was given an intraperitoneal saline injection on Day 5 of the experiment. FSO treatment ameliorated 5‐FU‐induced intestinal mucositis. On immunohistologic examination, FSO suppressed significantly the activation of NF‐κB and expression of IL‐β and TNF‐α of the harvested intestinal tissue. The reduced dose FSO (mucositis FSO‐R) was as effective as the full dose (mucositis FSO) in suppressing IL‐β and TNF‐α production, but was not as effective as the full dose in suppressing NF‐κB. On light microscopy, FSO attenuated significantly 5‐FU‐induced anomalies, such as the reduction of intestinal villus length and Goblet cell count. The reduced dose FSO (mucositis FSO‐R) was as effective as the full dose (mucositis FSO) in restoring villus length, but was not as effective as the full dose in restoring Goblet cell count. The findings of the study suggest that FSO inhibits 5‐FU‐induced intestinal mucositis via modulation of mucosal inflammation.

## Graphical abstract

Fig seed oil has significant protective effects against experimental intestinal mucositis induced by 5‐fluorouracil in rats. This beneficial effect of fig seed oil may be attributed to the antioxidant properties of both alpha linolenic acid and gamma tocopherol. Fig seed oil, as cold‐press natural plant seed oil, may serve as a valuable medicine for preventing and treating chemotherapy‐induced intestinal mucositis.

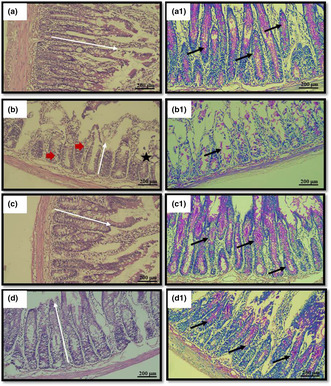

## 1 INTRODUCTION

Chemotherapy or radiotherapy is an effective method for treating malignant tumors. However, these treatments act not only on tumors but also on normal tissues, which eventually leads to various clinical side effects. Since the whole gastrointestinal tract is lined by cells that can rapidly regenerate, they are readily affected by those side effects. The resultant pathological condition, seen in both oral and intestinal mucosa is so‐called mucositis (Brown & Gupta, 2020). Histologically, there are widespread inflammatory cell infiltration, disappearance of intestinal crypts with villus atrophy, desquamation of mucosal cells, ulceration, and even focal bleeding (Thomsen & Vitetta, 2018). Clinical manifestations of mucositis include pain related to oral or intestinal ulceration, elevation of inflammatory markers in blood chemistry, diarrhea, rectal bleeding, and infection. They all affect regular nutrition and bowel habits, thereby reducing body weight. Mucositis frequently restricts the patient's capacity to tolerate treatment, thus resulting in premature interruption of the treatment. This also negatively affects the treatment success and quality of patients' lives. Patients afflicted with mucositis face twice the likelihood of developing infections and are four times more prone to mortality in comparison to those not affected by the condition (Brown & Gupta, 2020). Around 30%–40% of cancer patients treated with chemotherapy experience mucositis. This figure increases to 60%–85% for patients undergoing hematopoietic stem cell transplantation and nearly 90% for individuals with head and neck cancer undergoing combined radiotherapy and chemotherapy (Brown & Gupta, 2020; Kwon, 2016; Pulito et al., 2020; Thomsen & Vitetta, 2018).

The Multinational Association of Supportive Cancer Care in Cancer (MASCC) and the International Society of Oral Oncology (ISOO) recommend “basic oral care” as the most effective measure in all cancer groups to prevent mucositis in the guideline published in 2020 (Elad et al., 2020). However, many pharmacological and nonpharmacological applications are still being tried to control mucositis. Although many products, such as natural herbal/animal products, vitamins, minerals, etc., which have topical or systemic anti‐inflammatory and tissue healing properties, are not actively included in this guideline, they are still included in the appendices of this guideline as a potential curative and preventive measure (Yarom et al., 2019, 2020). Natural herbal products can improve the intestinal mucosal barrier, decrease enterocyte apoptosis, suppress inflammatory response and oxidant stress, and modulate gut microflora and the immune system. Many such products have been investigated in the 5‐fluorouracil (5‐FU)‐induced intestinal mucositis animal model, a well‐known and widely used animal model in the literature (Li et al., 2022).

Turkey is the leading producer of figs globally, contributing to 30% of the world's production. Figs, belonging to the mulberry family and scientifically known as *Ficus carica* L., are versatile fruits consumed raw, dried, canned, or preserved. They thrive in regions with hot, dry summers, and mild winters, such as Turkey, Egypt, Morocco, Spain, Greece, California, Italy, Brazil, and several other countries (Taş, 2019).

Fig contains up to 1500 seeds per fruit. Dry seeds contain about one‐third of oil by weight. According to the results of studies conducted mainly on figs from Aydın, a province in the west of Turkey, fig seed oil (FSO) contains a relatively high amount of alpha‐linolenic acid (ALA) (omega‐3 fatty acids) (Baygeldi et al., 2021; Duman & Yazici, 2018; Ergün & Bozkurt, 2020; Güven et al., 2019; Hssaini et al., 2020; Taş, 2019) and vitamin E (Baygeldi et al., 2021; Tarlacı, 2021), both of which are well‐known natural antioxidants. In the present study, we investigated the possible health protection effect of FSO as a whole food supplement. We tested its beneficial effects on the 5‐FU‐induced intestinal mucositis animal model.

## 2 MATERIALS AND METHODS

### 2.1 Laboratory animals and ethics approval

Twenty‐four randomly selected equal numbers of male and female albino Wistar adult rats weighing 200–216 g were used in our study. The research was carried out in the Multidisciplinary Experimental Animals Laboratory Unit of our institution. This study was approved by the institutional review board of Dokuz Eylul University (no.: 50/2019).

### 2.2 Housing conditions of laboratory animals

Macroenvironmental conditions of the room in which laboratory animals are housed are as follows: room temperature: 22 ± 2°C; relative humidity: 50 ± 10%; 12:12‐h day–night cycle (automatic lighting 07.00–19.00 daytime); light intensity: 150–200 lux; room ventilation: 8–10 changes/hour with a sound level of up to 70 dB. There were three animals of the same sex per cage in the experimental setup to control for any potential gender‐specific effects on the observed outcomes. They reached drinking water ad libitum with pellet feed specific to rats.

### 2.3 Animal groups

After allowing 1 week to adapt to macro‐ and microenvironments, all the animals were randomly divided into four groups. There were three male and three female rats in each group.

The same researcher performed oral administration of saline or FSO using a rat‐compatible oral gavage apparatus at the same time every day. Body weight measurements were performed and recorded on the groups' 0th, 5th, 8th, and 11th (sacrification) days. The same researcher performed an intraperitoneal injection of 5‐FU with a 23‐gauge needle. The drug dosage was individually adjusted according to rats' body weights. (Barros et al., 2018; Generoso et al., 2015; Mashtoub et al., 2013).

All the animals in the four groups had a normal standard diet and water throughout the experimental period, which lasted 11 days. Mucositis group: They were treated with a single oral administration of 0.6 mL saline daily throughout the experiment. Animals were also administered an intraperitoneal single doses of 300 mg/kg 5‐FU (Koçak, Turkey) on the fifth day of the study to trigger intestinal mucositis. We chose to perform sacrifice in the late period (sixth day after mucositis triggering) in order to better see the total protective and therapeutic effect of FSO, because earlier histological examination may miss the therapeutic effect that will emerge over time. This is also preferred in the literature (Barros et al., 2018; Generoso et al., 2015).

Mucositis FSO group: They were treated with a single oral administration of 0.6 mL FSO daily throughout the experiment. Animals were also administered intraperitoneal single doses of 300 mg/kg 5‐FU to trigger intestinal mucositis on the fifth day of the study.

Mucositis FSO‐R group (a mucositis group with reduced (R) FSO): they were treated with a single oral administration of 0.2 mL FSO +0.4 mL saline daily throughout the experiment. Animals were also administered intraperitoneal single doses of 300 mg/kg 5‐FU to trigger intestinal mucositis on the fifth day of the study.

Control group: Animals undergoing no oral intervention and intraperitoneal saline injection on the fifth day of the study.

### 2.4 Collection of tissue samples

On the 11th day of the experiment, rats were gently placed in an ether‐containing gas desiccator, and they were placed on the operating table under general anesthesia. After abdominal incision, the distal duodenal 4‐cm segment was taken from each rat (Akyuz et al., 2018; Al Asmari et al., 2016; Barros et al., 2018; Generoso et al., 2015; Whittaker et al., 2017; Yumusak et al., 2019).

### 2.5 Light microscopic examinations

The duodenum sections were first immersed in 10% buffered formaldehyde for 48–72 h to initiate fixation. Following this, the tissues underwent standard tissue processing procedures and were subsequently embedded in paraffin blocks. Using a microtome (RM 2135, Leica, Nussloch, Germany), serial sections with a thickness of 5 μm were obtained from these paraffin blocks. Subsequently, histological staining techniques, including hematoxylin–eosin, periodic acid–Schiff (PAS), and Masson trichrome, were employed on the serial sections to enable histomorphological examinations. For analysis, images were captured utilizing a light microscope (BX‐51, Olympus, Tokyo, Japan) equipped with a high‐resolution video camera (DP‐71, Olympus, Tokyo, Japan). Measurements were conducted utilizing the Image J program. Specifically, histomorphological parameters such as the length of villi in the hematoxylin–eosin staining and the quantification of Goblet cells in the PAS staining were calculated and analyzed.

### 2.6 Immunohistochemistry

The immunohistochemistry process utilized the streptavidin–biotin method. Initially, sections were placed on lysine‐coated slides and incubated overnight in a 60°C oven. Subsequently, they underwent deparaffinization through a xylene series followed by rehydration via an alcohol series. Antigen unmasking was achieved by treating the sections with 10‐mM citrate buffer at 95°C for 5 min. To block endogenous peroxidase activity, sections were outlined using a Dako pen (Dako, Denmark) and then incubated with 3% hydrogen peroxide in a 37°C oven for 15 min. Following this, the sections were treated with a normal serum‐blocking solution for 30 min and then incubated overnight in a humidity chamber (maintained at 30%–60%) with primary antibodies targeting IL‐β (Bioss‐USA, BS‐6319R, diluted 1:100), TNF‐α (Bioss‐USA, BS‐10802R, diluted 1:100), and NF‐κB (Bioss‐USA, BS‐0465R, diluted 1:100). On a subsequent day, the sections underwent washing with phosphate‐buffered saline and were then incubated with biotinylated IgG, followed by incubation with streptavidin–peroxidase conjugate (SensiTek HRP Anti‐Polyvalent Lab Pack, ScyTek Laboratories, Logan, United States). After three washes with phosphate‐buffered saline, the sections were treated with 3,3′‐diaminobenzidine (Roche Diagnostics, Switzerland) for 2 min to visualize immunoreactivity. Finally, the sections were mounted with entellan (Merck, Germany) after counterstaining with Mayer's hematoxylin (Sigma Aldrich, Germany) for 10 s.

### 2.7 Semiquantitative immunohistochemistry analysis

A grading system was employed to assess the quantity of IL‐β, TNF‐α, and NF‐κB positive cells in the tissue sections. The scoring criteria were as follows: 0 = absence of positive staining; 1 = mild positive staining; 2 = moderate positive staining; and 3 = intense positive staining evenly spread throughout the entire image. To ensure consistency in scoring, each section was evaluated by a histologist who was blinded to the experimental groups (Gencpinar et al., 2021).

### 2.8 Statistical analysis

The data obtained were subjected to statistical analysis using one‐way analysis of variance (ANOVA). SPSS version 25 was employed for the statistical analysis. Prior to parametric tests, the normality of distribution in each group was assessed using the Shapiro–Wilk test. Parametric data are presented as mean ± standard deviation, and intergroup comparisons were conducted using one‐way ANOVA. The homogeneity of variances was assessed using Levene's statistics. Post hoc analysis was performed using Bonferroni and Tukey's tests. For nonparametric tests, the Kruskal–Wallis test with Dunn's posttest was used. For further analysis (i.e., intergroup comparisons), the Mann–Whitney *U*‐test was conducted. A significance level of *p* < .05 was considered statistically significant.

## 3 RESULTS

**FIGURE 1:**
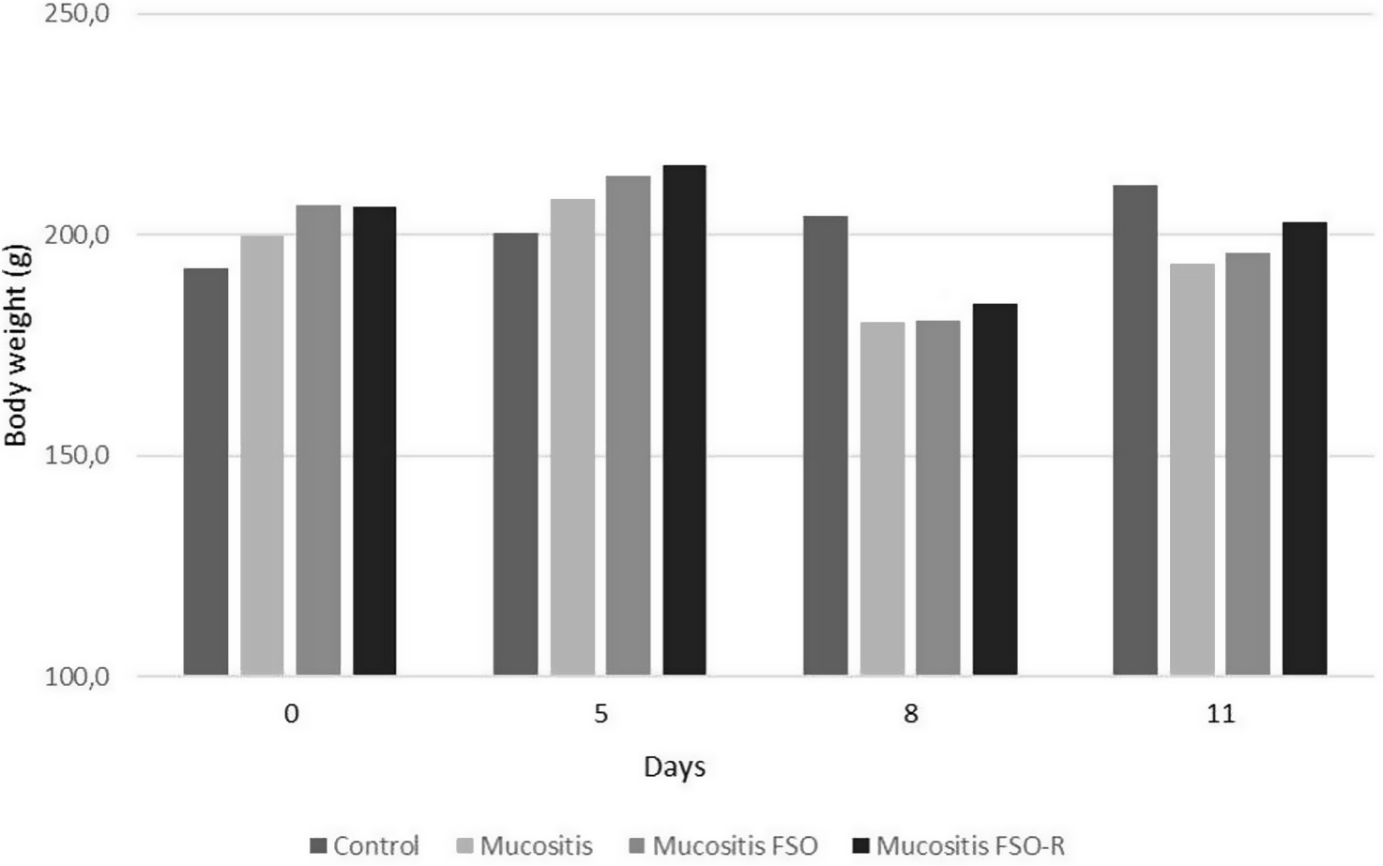
5‐FU administered on the fifth day significantly induced body weight loss in all three mucositis groups. Weight reduction reached the maximum level on the eighth day and resumed afterward.

During the entire experiment, compared with the other three groups, the rats in the control group had a steady increase in body weight. All the mucositis groups (mucositis, mucositis FSO, and mucositis FSO‐R) suffered weight loss after the triggering of mucositis on the fifth day; weight reduction reached maximum level on the eighth day. After that, those three groups gained weight up to the end of the experiment. One‐way ANOVA showed a statistical difference between the control and all mucositis groups only on the eighth day (Figure 1).

**FIGURE 2:**
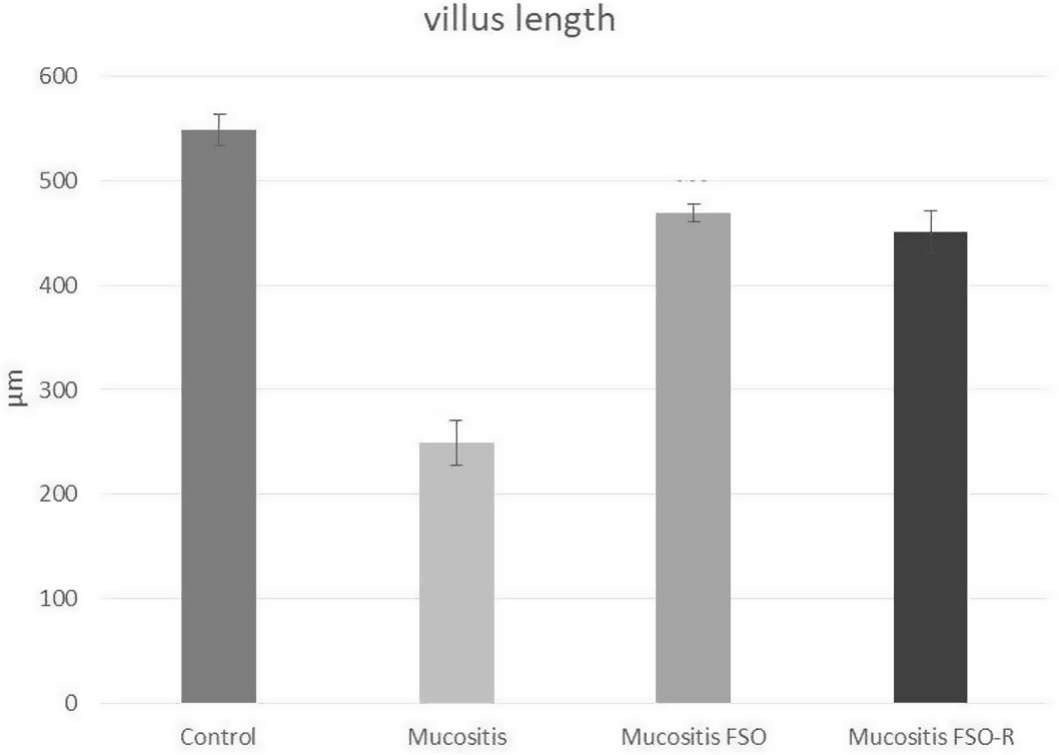
Evaluation of the villus length in 5‐FU‐induced mucositis. There was a statistical difference between the control and all three mucositis groups. To delineate the healing effect of FSO, we compared the mucositis group with mucositis FSO (*p* < .0001) and mucositis FSO‐R (*p* < .0001) groups and found statistically significant differences in terms of the villus length. There was no statistical difference between mucositis FSO and mucositis FSO‐R groups. So, even the reduced dose (three times less) of FSO improved the villus length nearly as much as the full FSO dose.

**FIGURE 3:**
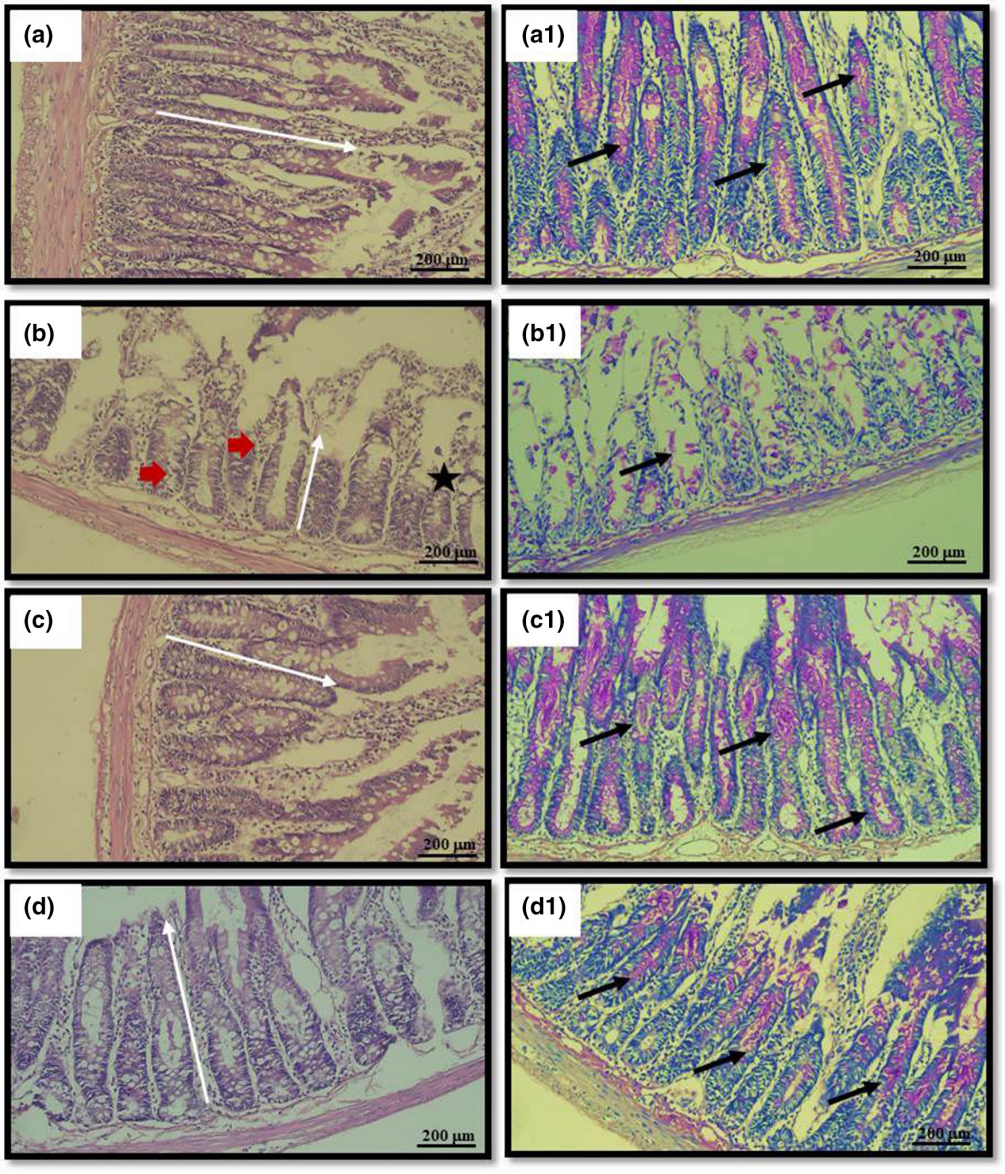
Histological samples from all the groups (the first column is hematoxylin + eosin, the second column is periodic acid–Schiff staining): (a‐a1) control group, (b‐b1) mucositis group, (c‐c1) mucositis FSO group, (d‐d1) mucositis FSO‐R group. The white arrows in the first column indicate the length of the villus sampled in the section. The black arrows in the second column indicate the distribution of Goblet cells. The red arrows and the star in the control group show epithelial shedding and vacuolization, respectively. Both are other typical features of mucosal damage.

Assessing the villus length in 5‐FU‐induced mucositis provides insight into the overall severity of mucosal damage. For each histologic sample, 20 villi were selected and measured, and the mean villus length was subsequently calculated. The distribution of villus length was normal in every group, and the variances were also homogeneous. One‐way ANOVA showed that there was a statistical difference between the control and all three mucositis groups in pairwise comparison. Significance levels were as follows; *p* < .001(control vs. mucositis), *p* < .023 (control vs. mucositis FSO), and *p* < .004 (control vs. mucositis FSO‐R). This indicated that full recovery in villus length does not yet occur 6 days after triggering mucositis even under the protection of FSO. To further delineate the healing effect of FSO, we compared the mucositis group with mucositis FSO (*p* < .0001) and mucositis FSO‐R (*p* < .0001) groups and found statistically significant differences in terms of the villus length. There was no statistical difference between the mucositis FSO and mucositis FSO‐R groups. So, 5‐FU triggering reduced the villus length in all the mucositis groups, with maximum reduction in the mucositis group. Premedication via oral FSO administration markedly alleviated the villus length reduction, which was observed in the mucositis FSO and mucositis FSO‐R groups. An important note is that even the reduced dose (three times less) of FSO improved the villus length nearly as much as the full FSO dose which was proven statistically (Figures 2 and 3).

**FIGURE 4:**
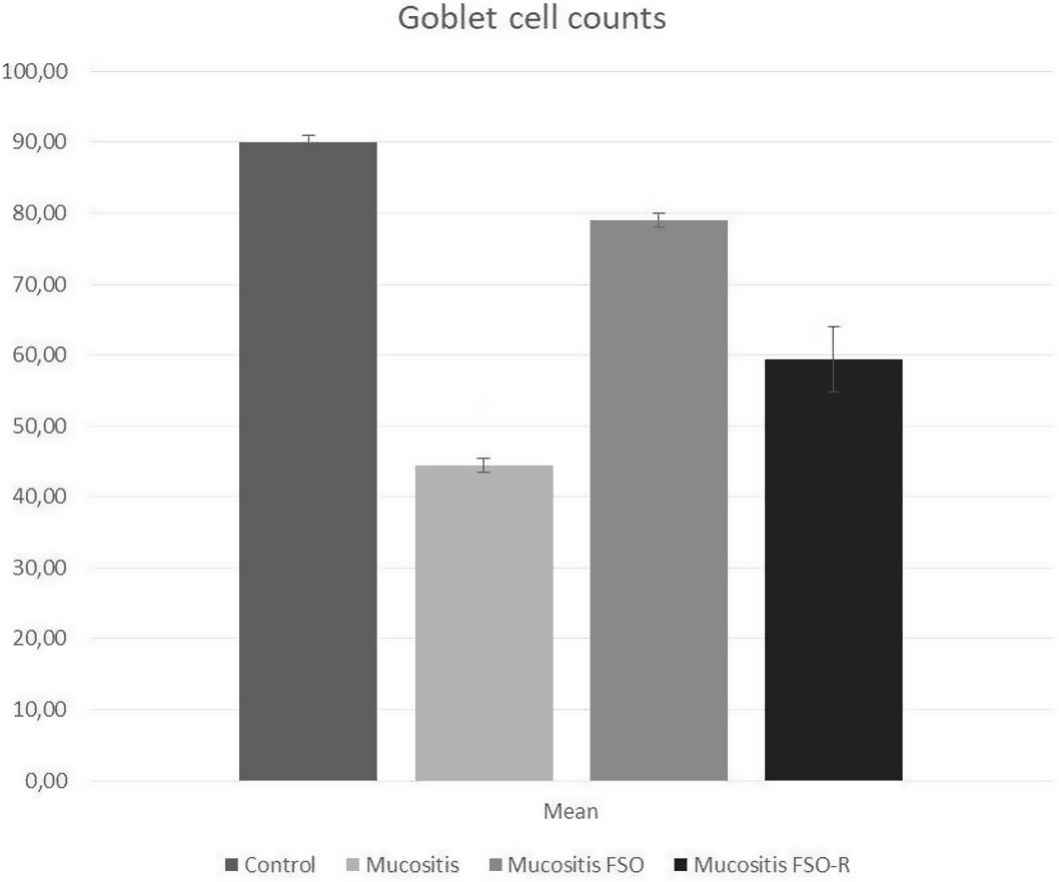
Evaluation of the Goblet cell counts in 5‐FU‐induced mucositis A statistically significant difference was observed between the control and mucositis and mucositis FSO‐R groups. No difference was found between the control and mucositis FSO groups. To further delineate the healing effect of FSO, we compared the mucositis group with two FSO groups and found significant difference in only one comparison (mucositis vs. mucositis FSO, *p* < .014) in terms of the Goblet cell counts. So, premedication with oral FSO leads to a nearly full recovery of Goblet cell count, which was proven statistically in the mucositis FSO group (i.e., full dose of FSO) only.

Assessing the Goblet cell count on histologic sample provides insight into the overall severity of mucositis. The obtained data were normally distributed. One‐way ANOVA showed that there was a statistical difference between the control and two mucositis groups in pairwise comparison. Significance levels were as follows; *p* < .001 (control vs. mucositis) and *p* < .035 (control vs. mucositis FSO‐R). No difference was found between the control and mucositis FSO groups. This indicated that nearly full recovery in Goblet cell count does occur 6 days after triggering mucositis under protection of only full dose of FSO (i.e., mucositis FSO). To further delineate the healing effect of FSO, we compared the mucositis group with two FSO groups, and found significant difference in only one comparison (mucositis vs. mucositis FSO, *p* < .014) in terms of the Goblet cell counts. So, 5‐FU triggering reduced the Goblet cell count in all mucositis groups, with maximum reduction in the mucositis group. Premedication via oral FSO administration markedly alleviated the Goblet cell reduction, leading to a nearly full recovery of Goblet cell count, which was proven statistically in only the mucositis FSO group. A reduced dose (three times less) of FSO improved Goblet cell counts slightly, not reaching a statistical difference (Figures 3 and 4).

Assessing the IL‐β, TNF‐α, and NF‐κB in immunohistochemistry provides insight into the inflammation level. The Mann–Whitney *U*‐test result showed that there was a statistical difference between the mucositis and mucositis FSO groups (*p* < .01), and between the mucositis and mucositis FSO‐R groups (*p* < .01) in terms of the IL‐β values. No difference was found between the mucositis FSO and mucositis FSO‐R. So, premedication via oral FSO administration markedly suppressed the IL‐β production, which was observed in both FSO groups. An important note is that even the reduced dose (three times less) of FSO suppressed the IL‐β production nearly as much as the full FSO dose which was proven statistically. There was a statistically significant difference between the mucositis and mucositis FSO groups (*p* < .005), and between the mucositis and mucositis FSO‐R groups (*p* < .005) in terms of the TNF‐α values. No difference was found between the mucositis FSO and mucositis FSO‐R. So, premedication via oral FSO administration markedly suppressed the TNF‐α production, which was observed in both FSO groups. An important note is that even the reduced dose (three times less) of FSO suppressed the TNF‐α production nearly as much as the full FSO dose which was proven statistically. There was a statistical difference between the mucositis and mucositis FSO groups (*p* < .005), and between the mucositis and mucositis FSO‐R groups (*p* < .05), and mucositis FSO and mucositis FSO‐R groups (*p* < .05) in terms of the NF‐κB. So, premedication via oral FSO administration markedly suppressed the NF‐κB production, which was observed in both FSO groups. The reduced dose (three times less) of FSO, however, did not suppress the NF‐κB production as powerful as the full FSO dose.

**FIGURE 5:**
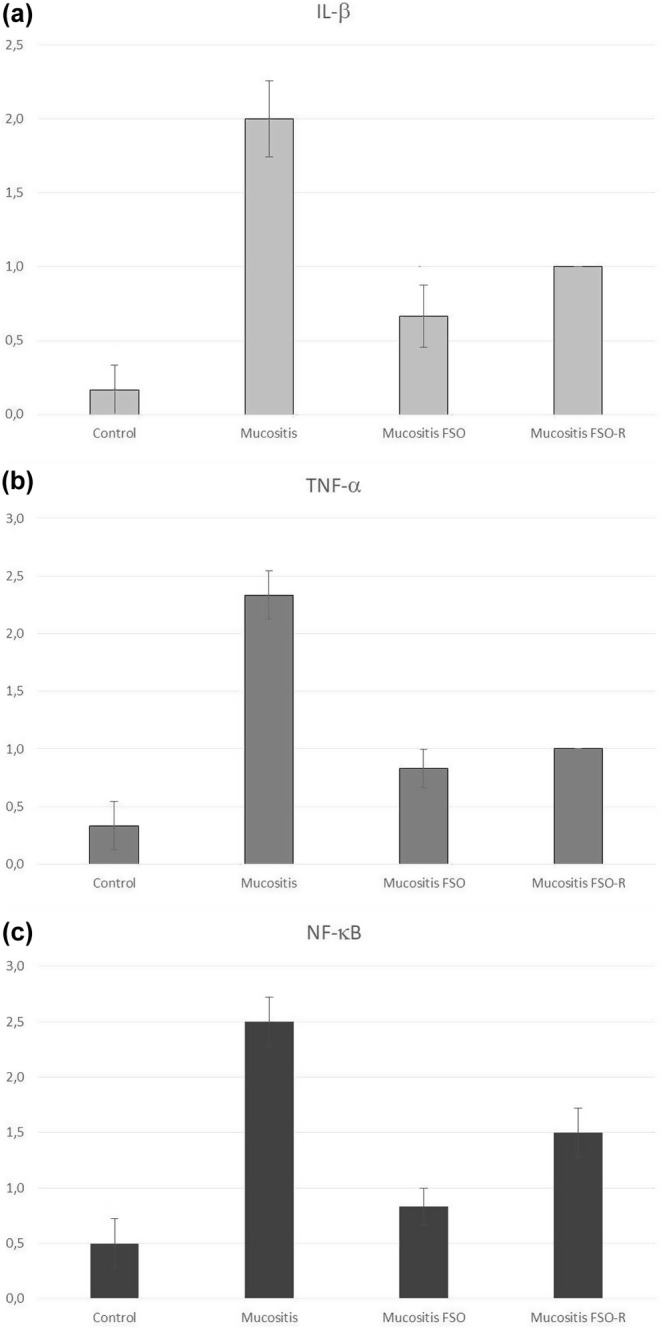
Evaluation of the expression of three well‐known inflammation markers, IL‐β, TNF‐α, and NF‐κB, in the harvested intestinal specimens. (a) There was a statistical difference between the mucositis and mucositis FSO groups (*p* < .01), and between the mucositis and mucositis FSO‐R groups (*p* < .01) in terms of the IL‐β values. No difference was found between the mucositis FSO and mucositis FSO‐R. (b) There was a statistically significant difference between the mucositis and mucositis FSO groups (*p* < .005), and between the mucositis and mucositis FSO‐R (*p* < .005) groups in terms of the TNF‐α values. No difference was found between the mucositis FSO and mucositis FSO‐R. (c) A statistical difference was observed between the mucositis and mucositis FSO groups (*p* < .005), between the mucositis and mucositis FSO‐R groups (*p* < .05), and between the mucositis FSO and mucositis FSO‐R groups (*p* < .05) in terms of the NF‐κB values. The reduced dose (three times less) of FSO, however, did not suppress the NF‐κB production as powerful as the full FSO dose.

5‐FU triggering resulted in increased production of all three markers in all mucositis groups, with maximum increase in the mucositis group. In the mucositis FSO and mucositis FSO‐R groups, premedication via oral FSO administration markedly alleviated the marker increase, meaning a dampened inflammatory response. Notably, even a reduced dose of FSO (three times less) suppressed the production of IL‐β and TNF‐α and improved inflammation in harvested tissue almost to the extent of the full FSO dose. However, the suppression of NF‐κB in the reduced dose (mucositis FSO‐R group) was not as potent as in the full FSO dose (mucositis FSO group) as shown in Figure 5. This suggests that while a reduced dose of FSO is effective in the suppression of IL‐β and TNF‐α, and mitigating inflammation, its impact on NF‐κB suppression may be less pronounced compared to the full dose.

## 4 DISCUSSION

In our study conducted in rat models, histologic examination revealed a reduction in the villus length and Goblet cell count in the harvested tissue, typical morphological features of mucosal damage. Immunohistologic examination revealed increased expression of inflammatory cytokines, representing increased inflammatory reaction associated with mucosal damage. Premedication with oral FSO significantly reversed both histologic and immunohistologic changes. Notably, the beneficial effect of the reduced (three times less) dose was nearly as powerful as the full dose of FSO.

5‐FU induction resulted in weight loss in the animal groups. This weight loss in all three mucositis groups may have occurred due mainly to the negative effect of 5‐FU on appetite, as well as mucositis in the intestine. The animals in all three mucositis groups regained their weight in the following days; this might be related to the subsiding destructive effect of a single dose of 5‐FU, rapid regeneration of tissues, and recovery of appetite. Indeed, no positive effect of FSO on weight was detected throughout the entire experiment (see Figure 1). The decrease in appetite after 5‐FU induction was the most important reason why we preferred gavage as the method of FSO administration. Thus, all experimental animals received equal doses of FSO, regardless of appetite status.

### 4.1 FSO composition

The FSO contains a relatively high amount of ALA (alpha‐linolenic acid). The ALA content ranges between 30% and 42%, with total polyunsaturated fatty acid (PUFA) contents of 68% and 88% (Baygeldi et al., 2021; Duman & Yazici, 2018; Ergün & Bozkurt, 2020; Güven et al., 2019; Hssaini et al., 2020; Taş, 2019). The ALA content was reported as 53% in figs obtained from the California region (Jeong & Lachance, 2001). In general, edible plant seed oils are accepted as a poor source of omega‐3 fatty acids since they have <1%–1.5% ALA (Orsavova et al., 2015). Linseed oil has the highest ALA content among edible plant oils; around 50% of all fatty acids in flaxseed are ALA (Bayrak et al., 2010). Due to its roughly 35%–40% ALA content, FSO is the only edible oil similar to linseed oil.

Another distinctive feature of FSO is that it contains a high amount of tocopherol (vitamin E); it was reported as 325 mg/100 g in a study (Baygeldi et al., 2021) and 404 mg/100 g in another study (Tarlacı, 2021). Plant seeds are a well‐known source of tocopherol. Their total tocopherol levels are well below 100 mg/100 g, although wheat germ and pumpkin oil are exceptions because they have as high as 250–300 mg/100 g tocopherol (Shahidi & Camargo, 2016). Tocopherols are present in four homologs (alpha, beta, delta, and gamma), distinguished by variations in the number and position of methyl groups within their chemical structures. More than 95% of total tocopherol is gamma tocopherol in FSO (Baygeldi et al., 2021; Güven et al., 2019; Tarlacı, 2021) and pumpkin seed oil (Shahidi & Camargo, 2016). Therefore, FSO differs from all edible plant seed oils containing tocopherol in terms of total tocopherol content (by far the highest) and tocopherol type (i.e., gamma).

### 4.2 Pathophysiology of mucositis

Mucositis development involves a series of sequential stages, as outlined by Pulito et al. (2020). The initiation phase is triggered directly by radiotherapy or chemotherapy, inducing initial intracellular damage. This phase is characterized by the production of reactive oxygen species (ROS), accompanied by DNA damage, ultimately resulting in the death of epithelial cells. The release of endogenous damage‐associated pattern molecules (DAMPs) from these dead cells further promotes the transcription of numerous genes implicated in mucositis development. Notably, NF‐κB serves as the primary transcriptional mediator, regulating over 200 genes associated with proinflammatory cytokines (such as IL‐β and TNF‐α), cell adhesion molecules, stress responders, and cytokine modulators. This process also involves the activation of other transcription factors, kinases, and metalloproteinases. Concurrently with the activation of various inflammatory pathways, the initial damage is exacerbated through positive feedback loop mechanisms. This, in turn, hampers the cell cycle, recruits leukocytes, and sustains NF‐κB activity, thereby initiating the breakdown of cellular and intercellular integrity. The creation of breaches in the submucosa permits numerous microorganisms, typically symbiotic inhabitants of healthy mucosa, to infiltrate this vulnerable tissue. This invasion leads to inflammatory cell infiltration, triggering the release of additional proinflammatory cytokines, which further enhance the expression of proapoptotic mediators. Ultimately, this process results in erosion and loss of crypts and villi. At this stage, both mucosal and submucosal integrity are compromised, causing patients to experience pain and potentially necessitating caregiver intervention (Pulito et al., 2020).

### 4.3 Effects of Omega‐3 on mucositis

While most fatty acids can be synthesized within the human body, essential fatty acids like linolenic and linoleic acids must be obtained from the diet. ALA undergoes limited conversion in the body into eicosapentaenoic acid (EPA). Following elongation and desaturation, EPA is further converted into docosahexaenoic acid (DHA) through β‐oxidation. Seafood primarily serves as another significant source of EPA and DHA. ALA, EPA, and DHA are categorized as omega‐3 polyunsaturated fatty acids (PUFAs). Numerous positive biological effects of omega‐3 PUFAs in preventing and treating various pathologic conditions have been reported; a detailed discussion can be found in the literature. There are few experimental animal studies on the roles of EPA and DHA in preventing and treating intestinal mucositis. In those experiments, fish oil (Fukatsu et al., 2008; Generoso et al., 2015; Ghosh et al., 2013; Torres et al., 2008), animal oil (Mashtoub et al., 2013, 2016), and commercial capsule (Abou‐Elez Gawish et al., 2013; Koppelmann et al., 2013) have been used as an omega‐3 fatty acid source. Overall, it has been concluded that omega‐3 PUFAs may have the potential to reverse intestinal microbial dysbiosis, attenuate intestinal inflammation, reduce oxidative stress, and consequently reverse mucosal damage in the gut damaged by chemotherapy (Zhang et al., 2019).

Similar to EPA and DHA, the less‐studied omega‐3 oil, ALA, also suppresses oxidative and immunological stress and has anti‐inflammatory effects with improved tissue healing in an animal model of colitis (Hassan et al., 2010; Kangwan et al., 2021; Wen et al., 2019).

### 4.4 Effects of tocopherol on mucositis

Until recently, the focus of clinical and experimental research primarily centered on alpha‐tocopherol as vitamin E, while other isoforms of tocopherols were disregarded. However, gamma and delta‐tocopherol, which differ from alpha‐tocopherol in the degree of methylation and the position of the methyl group on the chromanol ring, have been found to possess antioxidant properties and the ability to scavenge reactive nitrogen species alongside ROS, as noted by Abraham et al. (2019). This discovery has led to an increased interest in gamma‐tocopherol due to its superiority over the alpha isoform. Recent experimental studies (Guan et al., 2012; Jiang et al., 2000; Shin et al., 2017) and clinical investigations (Burbank et al., 2018; Deveraj et al., 2008; Tucker, 2017; Vucinic et al., 2010) across various diseases and pathological conditions have consistently demonstrated the superior efficacy of gamma tocopherol compared to alpha‐tocopherol.

Tocopherols cannot be synthesized in the human body and must be taken from foods such as plant seed oils, nuts, and vegetables. It plays essential roles in vision, reproduction, and maintaining blood, brain, and skin health. Numerous positive biological effects of tocopherols have been reported in laboratory and human studies, such as anti‐inflammatory, antiproliferative, and antiapoptotic effects (Ungurianu et al., 2021). One capsule a day of vitamin E (usually alpha‐tocopherol, natural, or synthetic) is recommended for health promotion, but up to three capsules (about 1000 mg) a day could be ingested safely in case of therapeutic usage (Ungurianu et al., 2021). A detailed discussion of the indication or dosing of tocopherol is beyond the scope of this article. There are few experimental animal studies on the beneficial roles of tocopherol in preventing and treating intestinal mucositis (Al Asmari et al., 2016; Yumusak et al., 2019). Chemotherapy‐induced mucositis may involve the entire gastrointestinal mucosa; oral involvement is called oral mucositis. The effects of tocopherol in oral mucositis have been the subject of several clinical studies. A recent meta‐analysis has shown that topical (not oral) vitamin E is effective in preventing and treating oral mucositis in human subjects (Chaitanya et al., 2017). Similar beneficial effects of topical tocopherol have also been reported in an animal model of oral mucositis (Cuba et al., 2016). Thus, the following conclusion can be reached by making an analogy from the experimental and clinical articles on the effects of vitamin E on oral mucositis: tocopherols could be capable of reverting gut damage (including intestine) created by chemotherapy‐induced mucositis.

### 4.5 FSO dosing in mucositis

Rats can be fed up to 1 mL each time with gavage without any problems. In our study, we chose 0.6 mL as the starting dose. This corresponds to approximately 3 mL/kg/day of oil and 12 mg/kg/day of tocopherol. When this is adapted to humans, a dose of 750–1000 mg/day of vitamin E (in about 180–250 mL of oil) could be calculated. This calculated human dose is comparable to the recommended dose for therapeutic use of vitamin E (Ungurianu et al., 2021).

When the average ALA ratio is accepted as 35%, FSO contains about 300 mg/mL ALA, corresponding to approximately 900 mg/kg/day in rats. When this is adapted to humans, a dose of 60–80 g/day of ALA (in about 180–250 mL of oil) could be calculated. In human studies, 1–4 g/day is recommended when omega‐3 oils (i.e., EPA and DHA) are taken for health promotion and disease prevention. It was reported that the daily dose could be safely increased up to 7 g/day (Zhang et al., 2019). Overall, the human dose of ALA calculated from our study is higher than the recommended limits. On the other hand, three times reduced FSO dose (approximately 60–80 mL of oil) corresponds to about 250–350 mg/day of vitamin E and 20–25 g/day of ALA in a human dose equivalent. For vitamin E (Ungurianu et al., 2021) and ALA (Allman et al., 1995; Kelley et al., 1993), those doses seem to be within human limits. Regarding volume, three times reduced FSO equates to about four to five tablespoons of oil per day for humans.

### 4.6 Monitoring the effect of FSO in mucositis

In our immunohistochemical analysis, NF‐κB, IL‐β, and TNF‐α were utilized as markers of tissue injury to assess the impact of FSO on mucositis. NF‐κB serves as a redox‐sensitive transcription factor pivotal in inflammation, apoptosis, cell division, differentiation, and development (Wu & Kral, 2005). While in a resting state, NF‐κB remains localized in the cytoplasm, it translocates into the nucleus under stress conditions, thereby regulating the expression of numerous genes. NF‐κB activation leads to the accumulation of various biologically active proteins and proinflammatory cytokines, including IL‐β and TNF‐α, ultimately contributing to gastrointestinal tract injury (Chang et al., 2012; Sonis, 2004). Induction of mucositis increased the expression of NF‐κB as well as IL‐β and TNF‐α as evident in immunohistochemical analysis (Figure 5), which is in accordance with the previous knowledge (Al Asmari et al., 2016; Hassan et al., 2010; Lessa et al., 2021; Zhang et al., 2019). Vitamin E (Calfee‐Mason et al., 2004; Cindrova‐Davies et al., 2007; Pal et al., 2013) and omega‐3 oils (Hassan et al., 2010; Lessa et al., 2021; Zhang et al., 2019) suppress the expression of NF‐κB together with cytokines, such as IL‐β and TNF‐α, which are assumed to be related to their antioxidant potential.

The impact of FSO administration on the severity of chemotherapy‐induced intestinal mucositis has not been investigated before. The FSO we used in the study has an extraordinary feature; it contains relatively high levels of ALA (i.e., omega‐3 oil) and gamma‐tocopherol (i.e., vitamin E).

### 4.7 Limitation

The symbiotic bacteria residing in the intestine play a vital role in regulating nutrient metabolism and absorption, preserving epithelial equilibrium, and enhancing immune tolerance within the gut. After the induction of mucositis, intestinal microbial homeostasis is also disrupted. One limitation of this study is the need for more investigation of intestinal microbial dysbiosis. We created animal groups with three animals of the same sex (six in total). We know that hormonal variations can affect the animal's immune/inflammatory response, which may affect experimental outcomes; this is another limitation of our study. One another limitation of our study is that a single sample was taken only from the distal duodenum, instead of taking each three segments (duodenum, jejunum, and ileum) of the small intestine separately.

## 5 CONCLUSION

FSO significantly protects against experimental intestinal mucositis induced by 5‐FU in rats. This beneficial effect of FSO may be attributed to the antioxidant properties of both ALA and gamma‐tocopherol.

## AUTHOR CONTRIBUTIONS

**Nurten Alan:** Data curation (equal); funding acquisition (lead). **Nazan Tuna Oran:** Conceptualization (lead); investigation (lead); methodology (equal); supervision (lead). **Pınar Akokay Yılmaz:** Data curation (equal); formal analysis (equal); methodology (equal). **Aslı Çelik:** Data curation (equal); formal analysis (equal). **Osman Yılmaz:** Conceptualization (equal); data curation (equal); funding acquisition (equal); methodology (equal).

## FUNDING INFORMATION

The study was supported by the Scientific Research Project Office of Dokuz Eylul University (Project number: TSA‐2022‐2565).

## CONFLICT OF INTEREST STATEMENT

The authors declare that they do not have any conflict of interest.

## Data Availability

The data that support the findings of this study are available on request from the corresponding author.
